# Relationship between peroneus longus tendon graft thickness and anthropometric variables: a radiographic study using ultrasonography

**DOI:** 10.1186/s43019-024-00235-4

**Published:** 2024-10-14

**Authors:** Koray Kaya Kilic, Fırat Dogruoz, Omer Faruk Egerci, Murat Yuncu, Aliekber Yapar, Ozkan Kose

**Affiliations:** 1 Department of Radiology, Antalya Training and Research Hospital, Antalya, Turkey; 2 Department of Orthopedics and Traumatology, Antalya Training and Research Hospital, Varlık mah., Kazım Karabekir cd., Muratpasa, 07100 Antalya, Turkey

**Keywords:** Peroneus longus tendon, Anterior cruciate ligament, Anthropometry, Graft thickness, Tegner Activity Scale

## Abstract

**Background:**

This study aimed to evaluate the predictive value of anthropometric measurements for two-stranded peroneus longus tendon (PLT) graft thickness using ultrasonography

**Materials and methods:**

A prospective study was conducted on 204 healthy volunteers (102 males and 102 females) aged 18–40 years. Anthropometric measurements were recorded, including height, weight, body mass index (BMI), fibular length, calf circumference, and ankle circumference. The Tegner Activity Scale (TAS) was used to assess activity levels. PLT cross-sectional area (CSA) was measured using ultrasonography. Two-stranded PLT graft thickness was calculated using the previously reported formula by Luo et al. A thickness of less than 8 mm of PLT graft was accepted as an insufficient autograft for anterior cruciate ligament reconstruction (ACLR). Correlation and regression analyses were performed to identify predictors of two-stranded PLT graft thickness. Receiver operating characteristic (ROC) analysis was performed to establish the best threshold values.

**Results:**

Males had a significantly greater PLT CSA (0.17 ± 0.03 cm^2^) and predicted two-stranded PLT graft thickness (8.1 ± 0.6 mm) compared with females (0.15 ± 0.03 cm^2^ and 7.5 ± 0.6 mm, respectively; *p* < 0.001 for both). Correlation analysis revealed that two-stranded PLT graft thickness positively correlated with height, weight, BMI, fibular length, calf circumference, ankle circumference, and Tegner Activity Scale in both genders, with stronger correlations observed in females. The logistic regression model identified height and calf circumference as significant predictors of sufficient two-stranded PLT graft thickness (≥ 8 mm) in males, while calf circumference and the TAS were significant predictors in females. ROC analysis demonstrated that calf circumference and the TAS had acceptable discriminatory abilities in females, with 36.25 cm and ≥ 4 cutoff points, respectively. However, no anthropometric variables in males exhibited strong discriminatory abilities for predicting two-stranded PLT graft thickness

**Conclusions:**

Calf circumference and the TAS are significant predictors for two-stranded PLT autograft thickness in females. However, no anthropometric variables in males could be used strongly for prediction. These anthropometric measurements can assist in preoperative planning and decision-making, potentially improving ACLR outcomes by ensuring adequate graft thickness in females.

*Level of evidence:* Level II prospective study

## Introduction

Anterior cruciate ligament (ACL) injuries are common and often require surgical intervention to restore knee stability and function. The choice of graft for ACL reconstruction (ACLR) plays a pivotal role in the success of the surgery and patient outcomes. Autografts such as bone patellar tendon bone, hamstring, and quadriceps tendon are the most commonly preferred graft options [1, 2]. Adequate graft thickness is critical, as insufficient diameter is associated with higher rerupture rates. A minimum graft thickness of 8 mm is generally recommended for ACLR [3, 4]. Preoperative knowledge of graft thickness is beneficial for surgical planning and patient counseling. Anthropometric measurements offer a noninvasive, cost-effective way to predict graft size preoperatively. Significant correlations have been demonstrated between parameters such as height, weight, and BMI and autograft dimensions [5]. These findings support anthropometric measurements to improve optimal graft selection and surgical outcomes and reduce complications.

The peroneus longus tendon (PLT) has recently gained attention as an alternative graft option due to its favorable characteristics, including sufficient length, thickness, and strength, with minimal donor site morbidity [6, 7]. However, consistently reaching the recommended 8 mm thickness with PLT grafts remains challenging. Previous studies reported that a significant number of female patients had PLT graft thickness below 8 mm, highlighting the need for reliable preoperative identification of patients at risk for inadequate graft thickness [8, 9]. Previous studies have explored the estimation of PLT thickness in relation to anthropometric parameters, often using small sample sizes and combining male and female data despite known gender differences in PLT thickness [8–15] (Table 1). However, these studies frequently relied on correlation analysis, which may not provide strong predictive value for clinical practice. There is a need for larger studies focusing on gender-specific analyses and more robust predictive methodologies.

**Table 1:** Previous studies investigated the relationship between anthropometric variables and PLT autograft thickness

Author	Year	Country	Patients	Graft preparation	Graft Ø (mm ± SD)	Anthropometric measures	Correlation analysis	Regression analysis
Song et al.	2018	China	156 (118 M, 38 F)	Four strands	8.3	Age, gender, height, weight, BMI, duration of injury, preinjury activity level	Age, gender, height, weight, BMI, duration of injury, preinjury activity level	Height, weight, and duration of injury
Rhatomy et al.	2019	Indonesia	39 (28 M, 11 F)	Not specified	8.56 ± 0.82	Gender, age, weight, height BMI	Gender Weight Height BMI	Not performed
Sakti et al.	2020	Indonesia	20 (17 M, 3 F)	Two strands	8.1 ± 0.8	Gender, age, weight, height, BMI, true leg length Shank circumference shank length	Weight, height, true leg length, shank length	Weight, height, true leg length, shank length
Ertilav	2021	Turkey	52 (38 M, 14 F)	Two strands	8.3 ± 0.5	Gender, age, weight, height BMI, leg length, distal/proximal leg diameter	Gender, weight, height BMI, leg length, distal/proximal leg diameter	Gender, weight, height BMI, leg length, distal/proximal leg diameter
Khan et al.	2021	India	52 (46 M, 6 F)	Four strands	9.3 ± 0.4	Age, weight, height, BMI, duration of injury	Weight, height	Weight, height
Luo et al.	2023	China	26	Two strands	Not specified	Weight, height, BMI, USG PLT CSA	Weight, height, USG PLT CSA	USG PLT CSA
Wierer et al.	2023	Germany	128 (46 F, 82 M)	AHPLT 4 strands	7.7 ± 0.8	Age, sex, height, weight, BMI, thigh and shank length, and thigh and shank circumference	Weight, height, BMI, shank circumference	Gender
Dar et al.	2023	India	34 (25 M, 9 F)	Four-strand graft	Male: 8.4 Female: 7.6	Age, sex, weight, height, BMI	Age, sex, weight, height, BMI	Not performed
Current study	2024	Turkey	204 (102 M, 102 F)	USG (PLT CSA)	Male: 8.1 ± 0.6 Female: 7.5 ± 0.6	Age, sex, weight, height, BMI, fibular length, calf circumference, ankle circumference, Tegner Activity Scale	Age, sex, weight, height, BMI, fibular length, calf circumference, ankle circumference, Tegner Activity Scale	Male: height, calf circumference with (weakly discriminatory) Female: calf circumference, Tegner activity scale

*M* male,* F* female,* SD* standard deviation,* BMI* body mass index,* USG* ultrasonography,* CSA* cross-sectional area,* PLT* peroneus longus tendon,* AHPLT* anterior half of the PLT,* Ø* diameter

This study aimed to evaluate the predictive value of anthropometric measurements for determining two-stranded PLT graft thickness using ultrasonography. It was hypothesized that individuals with higher physical activity levels and greater calf and ankle circumference would have thicker PLT tendons.

## Materials and methods

### Patients and study design

This study was conducted prospectively on healthy volunteer subjects in the authors’ institution between April 2024 and July 2024. Given that ACL injuries are more prevalent in younger patients, the study included an equal number of male and female patients between the ages of 18 and 40 years. All participants were screened to exclude those with lower-limb disorders, and a thorough physical examination was performed to rule out lower-extremity conditions with a particular focus on the foot and ankle. Subjects with a history of lower-limb injury, previous surgical operation, congenital or neuromuscular disease, or lower-limb abnormality were excluded from the study. All subjects were otherwise healthy and free of any known chronic disease. The research was conducted following the principles of the Declaration of Helsinki, and informed consent was obtained from each volunteer after a detailed explanation of the objectives and methods of the study. The local ethical committee approved the study protocol (approval number 2024.4/13).

### Sample size calculation

The sample size was calculated on the basis of previously published data to detect significant predictors of sufficient PLT graft thickness with an effect size estimated from relevant predictors in the literature, a significance level of 0.05, and a power of 0.80. Considering seven predictors in the logistic regression model and an average event rate of 40% of participants expected to have sufficient two-stranded PLT graft thickness [8], the required sample size was determined to be approximately 170 participants. To account for potential errors and ensure robustness, 204 participants (20% more) were included, equally divided between males and females.

### Anthropometric measurements

**Fig. 1:**
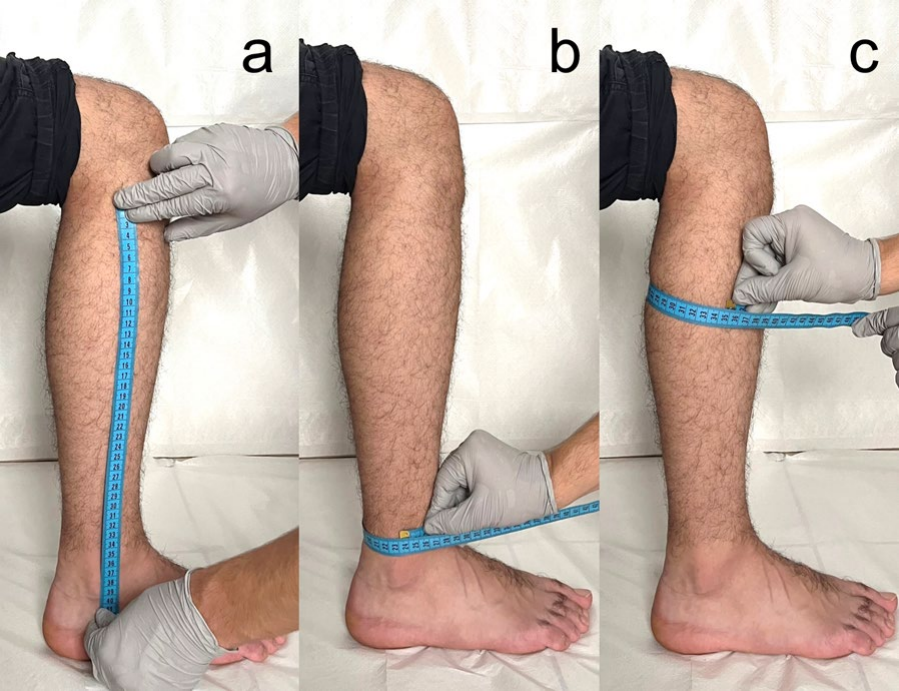
Anthropometric measurements conducted on the lower leg. **a** The distance between the fibular head and the tip of the lateral malleolus is recorded as the fibular length. **b** The ankle circumference is measured at the most distal point of the ankle, just above the joint line. **c** The calf circumference is measured at its widest point

Anthropometric measurements included height, weight, body mass index (BMI), fibular length, ankle circumference, and the widest calf circumference. The distance between the fibular head and the tip of the lateral malleolus was measured to ascertain the length of the fibula. The ankle circumference was measured at the most distal point of the ankle, just above the joint line. The circumference of the calf was measured at its widest point (Fig. 1). The same investigator conducted all measurements while the patient was seated with a measuring tape. Furthermore, the Tegner Activity Scale was employed to ascertain the subjects’ activity levels [16].

### Ultrasonographic measurements and calculation of two-stranded PLT graft

**Fig. 2:**
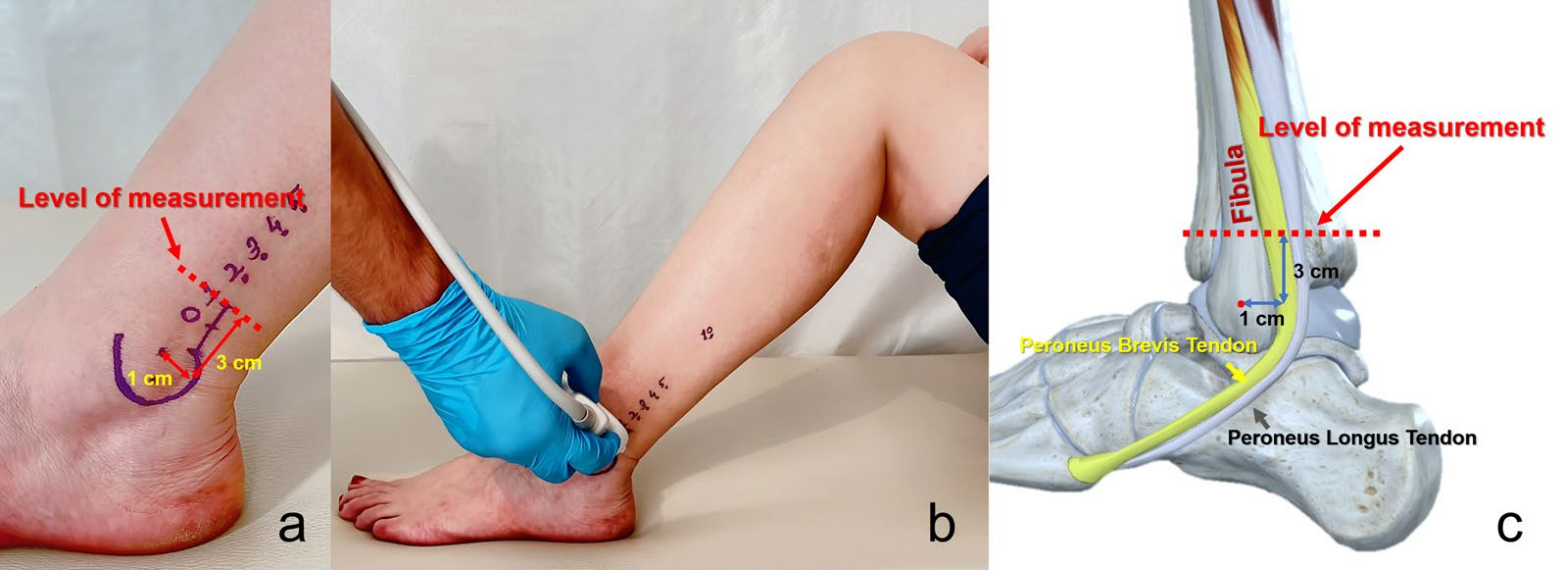
The USG examination and the level of measurement of the PLT and PBT tendons. **a** The subject is positioned in a supine position with the knee flexed at 90°. The midpoint of the lateral malleolus is marked, and the PLT harvest site is identified as 1 cm posterior and 2 cm proximal to this midpoint, designated as the zero point. Ultrasonographic images are obtained at 1 cm proximal to this point. **b** The USG probe is located perpendicular to the longitudinal axis of the tendons to obtain the axial image. **c** Anatomical illustration showing the level of measurement of the tendons

**Fig. 3:**
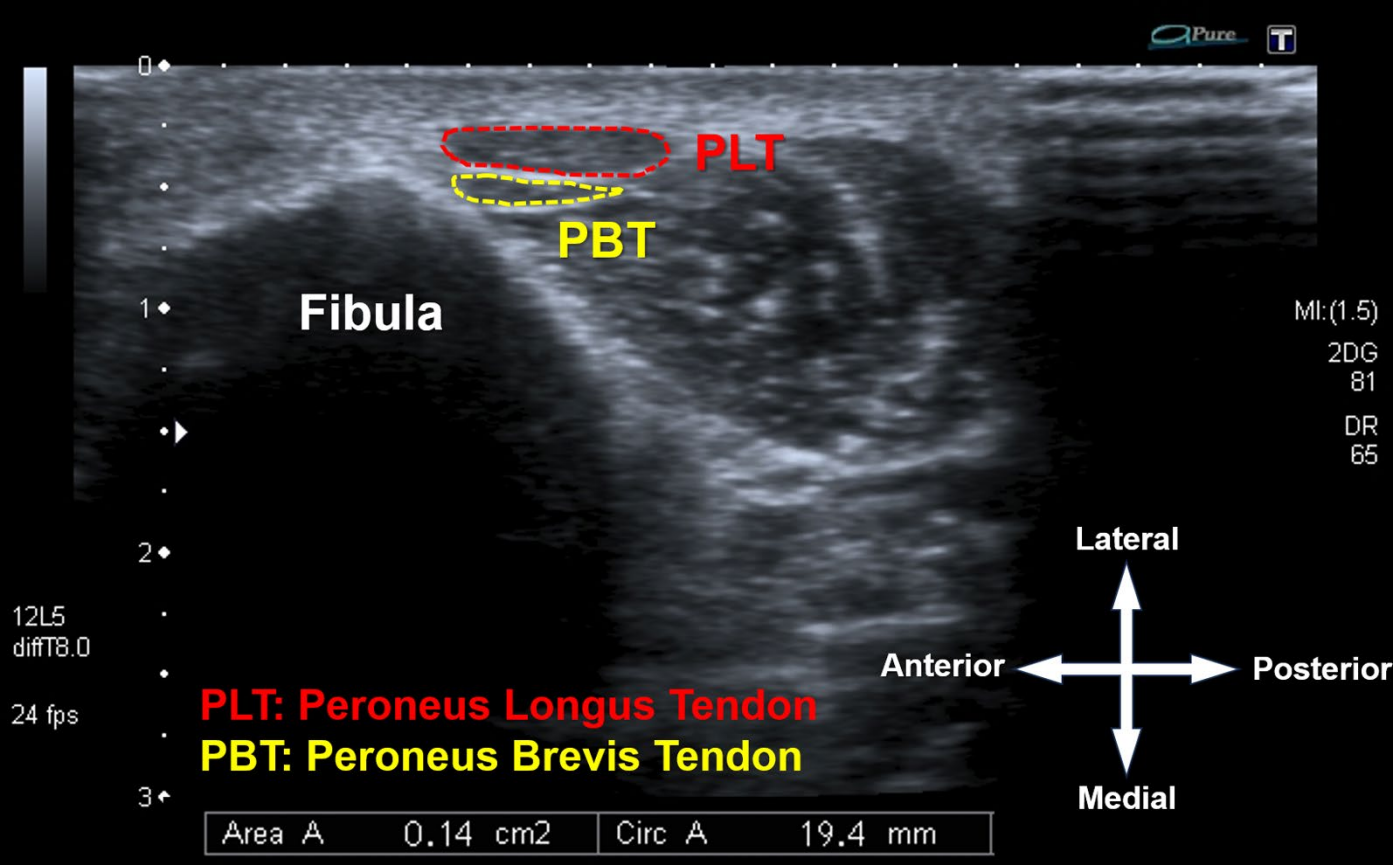
The USG image shows the axial mage of the PLT (red dotted area) and PBT (yellow dotted area)

Ultrasonographic examinations were performed using a Toshiba Aplio 500 system (Toshiba Medical System Corporation, Otawara, Japan) with a 14L5 (10 MHz) linear probe by a senior radiologist with over 10 years of experience in musculoskeletal ultrasonography (USG). Measurements and graft thickness calculations followed the methodology described by Luo et al. [11]. The subject was positioned supine with the knee flexed at 90°. The midpoint of the lateral malleolus was marked, and the PLT harvest site was identified as 1 cm posterior and 2 cm proximal to the midpoint, designated as the zero point. The PLT cross-sectional area (CSA) was measured at 1 cm proximal to this point, which best predicted the two-stranded tendon graft thickness [11]. Axial images were obtained perpendicular to the PLT and peroneus brevis tendon (PBT) longitudinal axis (Fig. 2), and the PLT tendon area was quantified using the ultrasound device’s area measurement application (Fig. 3). Intraobserver reliability was confirmed with an Intraclass correlation coefficient (ICC) of 0.984 [95% confidence interval (CI) 0.970–0.991] based on 40 PLT CSA measurements conducted twice with a 15-min interval. The two-stranded PLT graft thickness was predicted using the formula described by Leu et al. [11]: PLT thickness (mm) = 4.6 + 0.2 × PLT CSA (cm^2^).

### Statistical analysis

Continuous variables are presented as mean ± standard deviation, and categorical variables are presented as frequency and percentage. The Kolmogorov–Smirnov test was used to test the normality of data distribution. Comparative analysis of two independent groups was performed using the chi-square test, Mann–Whitney *U* test, and Student’s *t*-test. Pearson correlation test was used to analyze the correlation between the anthropometric measurements and the PLT length and thickness. Simple linear regression was used to determine the predictive value of anthropometric measurements on the two-stranded PLT graft thickness. Furthermore, ROC analysis was conducted on the parameters utilized to estimate adequate graft thickness (≥ 8 mm Ø), and the resulting thresholds were determined. Additionally, the sensitivity and specificity of these thresholds were calculated. Binary logistic regression (backward: Wald) analysis was conducted separately on male and female subjects to identify the significant predictors of a sufficient PLT graft (≥ 8 mm Ø). A *p*-value of less than 0.05 was deemed statistically significant.

## Results

The study included 204 participants, evenly divided between males (*n* = 102) and females (*n* = 102). The mean age was 29.4 ± 6.2 years. Bilateral measurements were taken for each participant, resulting in a total of 408 extremity measurements. Males were significantly taller and heavier than females, though BMI was similar between genders. The PLT CSA and predicted two-stranded PLT graft thickness were significantly larger in males compared to females. Detailed demographic and anthropometric characteristics are provided in Table 2​.

**Table 2:** Demographic and anthropometric characteristics of the patients

Variables (unit)	Male (*n* = 102)	Female (*n* = 102)	Total (*n* = 204)	*p*-Value
Age (years ± SD)	28.9 ± 6.2	29.9 ± 6.1	29.4 ± 6.2	0.237^1^
Height (cm ± SD)	177.5 ± 6.5	164.6 ± 5.5	171.1 ± 8.8	< 0.001^1^
Weight (kg ± SD)	80.1 ± 13.2	66.8 ± 14.3	73.4 ± 15.3	< 0.001^1^
Body mass index (kg/m^2^ ± SD)	25.3 ± 3.9	24.6 ± 4.9	25.0 ± 4.4	0.216^1^
Tegner Activity Scale (level)	4.6 ± 1.3	3.7 ± 1.1	4.1 ± 1.3	< 0.001^1^

	Male (*n* = 204)	Female (*n* = 204)	Total (*n* = 408)	
Fibular length (cm ± SD)	40.3 ± 2.0	36.8 ± 1.8	38.6 ± 2.6	< 0.001^1^
Calf circumference (cm ± SD)	37.3 ± 3.2	37.3 ± 4.4	37.5 ± 3.8	0.373^1^
Ankle circumference (cm ± SD)	22.7 ± 1.6	21.9 ± 2.2	22.3 ± 2.0	< 0.001^1^
PLT CSA (cm^2^ ± SD)	0.17 ± 0.03	0.15 ± 0.03	0.16 ± 0.03	< 0.001^1^
Two-stranded PLT Thickness (mm ± SD)	8.1 ± 0.6	7.5 ± 0.6	7.8 ± 0.6	< 0.001^1^
Height and weight adjusted PLT thickness (mm ± SD)	8.0 ± 0.2	7.6 ± 0.3	7.8 ± 0.4	< 0.001^1^

Data are presented as mean ± standard deviation. The *p*-value is for comparison between male and female subjects.^1^Mann–Whitney *U* test

*PLT* peroneus longus tendon,* CSA* cross-sectional area

The predicted two-stranded PLT graft thickness was below the 8 mm threshold in 43.6% of male legs and 70% of female legs, indicating a higher prevalence of insufficient grafts among females. Comparisons between subjects with sufficient and insufficient two-stranded PLT graft thickness revealed significant differences in most variables except age (Table 3).

**Table 3:** Comparison of anthropometric variables between subjects with sufficient and insufficient two-stranded PLT graft

Variables (unit)	Males			Females		
	PLT Ø ≥ 8 mm (*n* = 115, 56.4%)	PLT Ø < 8 mm (*n* = 89, 43.6%)	*p*-value	PLT Ø ≥ 8 mm (*n* = 59, 30.0%)	PLT Ø < 8 mm (*n* = 145, 70.0%)	*p*-value
Age (years ± SD)	28.8 ± 6.4	29.0 ± 5.9	0.709	30.4 ± 5.7	29.7 ± 6.3	0.588
Height (cm ± SD)	178.8 ± 5.9	175.9 ± 6.8	**0.006**	166.3 ± 5.4	163.9 ± 5.4	**0.004**
Weight (kg ± SD)	82.7 ± 13.8	76.8 ± 11.6	**0.001**	72.9 ± 13.3	64.3 ± 14.0	** < 0.001**
BMI (kg/m^2^ ± SD)	25.8 ± 4.2	24.8 ± 3.4	0.068	26.3 ± 4.4	23.9 ± 4.9	** < 0.001**
Fibular length (mm ± SD)	40.6 ± 1.8	40.0 ± 2.1	**0.026**	37.6 ± 1.6	36.4 ± 1.8	** < 0.001**
Calf circumference (mm ± SD)	38.4 ± 3.0	36.8 ± 3.3	** < 0.001**	39.5 ± 3.8	36.5 ± 4.3	** < 0.001**
Ankle circumference (mm ± SD)	23.1 ± 1.5	22.2 ± 1.5	** < 0.001**	22.9 ± 1.9	21.5 ± 2.2	** < 0.001**
Tegner Activity Scale (level ± SD)	4.8 ± 1.4	4.3 ± 1.1	**0.027**	4.3 ± 1.2	3.4 ± 0.9	** < 0.001**

The data are presented as mean and standard deviation, with *n* representing the number of legs. The Mann–Whitney *U* test is used for analysis. Bold p-values are statistically significant

Correlation analysis demonstrated significant relationships between two-stranded PLT graft thickness and various anthropometric variables across the study population (Table 4). The regression model identified height and calf circumference as significant predictors of sufficient two-stranded PLT graft thickness in males, while calf circumference and the Tegner Activity Scale were significant predictors in females. BMI was excluded from the model owing to high multicollinearity. The results of the regression analysis are summarized in Table 5**.**

**Table 4:** Correlation between the calculated PLT graft thickness and the demographic and anthropometric variables

Variables	Male (*n* = 204)	Female (*n* = 204)	Total (*n* = 408)
	PLT graft Ø	PLT graft Ø	PLT graft Ø
Age			
*ρ*	0.072	0.147	0.068
*p*-Value	0.308	**0.036**	0.168
Height			
*ρ*	0.255	0.293	0.445
*p*-Value	** < 0.001**	** < 0.001**	** < 0.001**
Weight			
*ρ*	0.400	0.443	0.513
*p*-Value	** < 0.001**	** < 0.001**	** < 0.001**
BMI			
*ρ*	0.305	0.377	0.346
*p*-Value	** < 0.001**	** < 0.001**	** < 0.001**
Fibular length			
*ρ*	0.128	0.330	0.404
*p*-Value	0.069	** < 0.001**	** < 0.001**
Calf circumference			
*ρ*	0.330	0.436	0.370
*p*-Value	** < 0.001**	** < 0.001**	** < 0.001**
Ankle circumference			
*ρ*	0.357	0.301	0.365
*p*-Value	** < 0.001**	** < 0.001**	** < 0.001**
Tegner Activity Scale			
*ρ*	0.212	0.348	0.362
*p*-Value	**0.002**	** < 0.001**	** < 0.001**

*BMI* body mass index, *PLT* palmaris longus tendon, *Ø* diameter

Bold values are statistically significant. Correlation coefficient: Pearson *ρ*, *n* number of feet

**Table 5:** Summary of binary logistic regression analysis

Male subjects									
Variables		B	S.E	Wald	d.f.	Sig.	Exp(B)	95% CI for Exp(B)	
								Lower	Upper
Step 7^a^	Height	0.064	0.024	6.957	1	**0.008**	1.066	1.016	1.117
	Calf circumference	0.145	0.048	9.076	1	**0.003**	1.156	1.052	1.270
	Constant	−16.487	4.531	13.242	1	**0.000**	0.000		

Female subjects									
Variables		B	SE	Wald	d.f.	Sig.	Exp(B)	95% CI for Exp(B)	
								Lower	Upper
Step 6^a^	Fibular length	0.201	0.107	3.534	1	0.060	1.222	0.991	1.507
	Calf circumference	0.144	0.042	11.510	1	**0.001**	1.155	1.063	1.255
	Tegner Activity Scale	0.620	0.146	17.990	1	**0.000**	1.859	1.396	2.476
	Constant	−16.212	3.849	17.744	1	**0.000**	0.000		

^a^Variable(s) entered on step 1: age, height, weight, BMI, fibular length, calf circumference, ankle circumference, Tegner Activity Scale

Bold *p*-values are statistically significant

Only the final steps of the analysis are presented

**Fig. 4:**
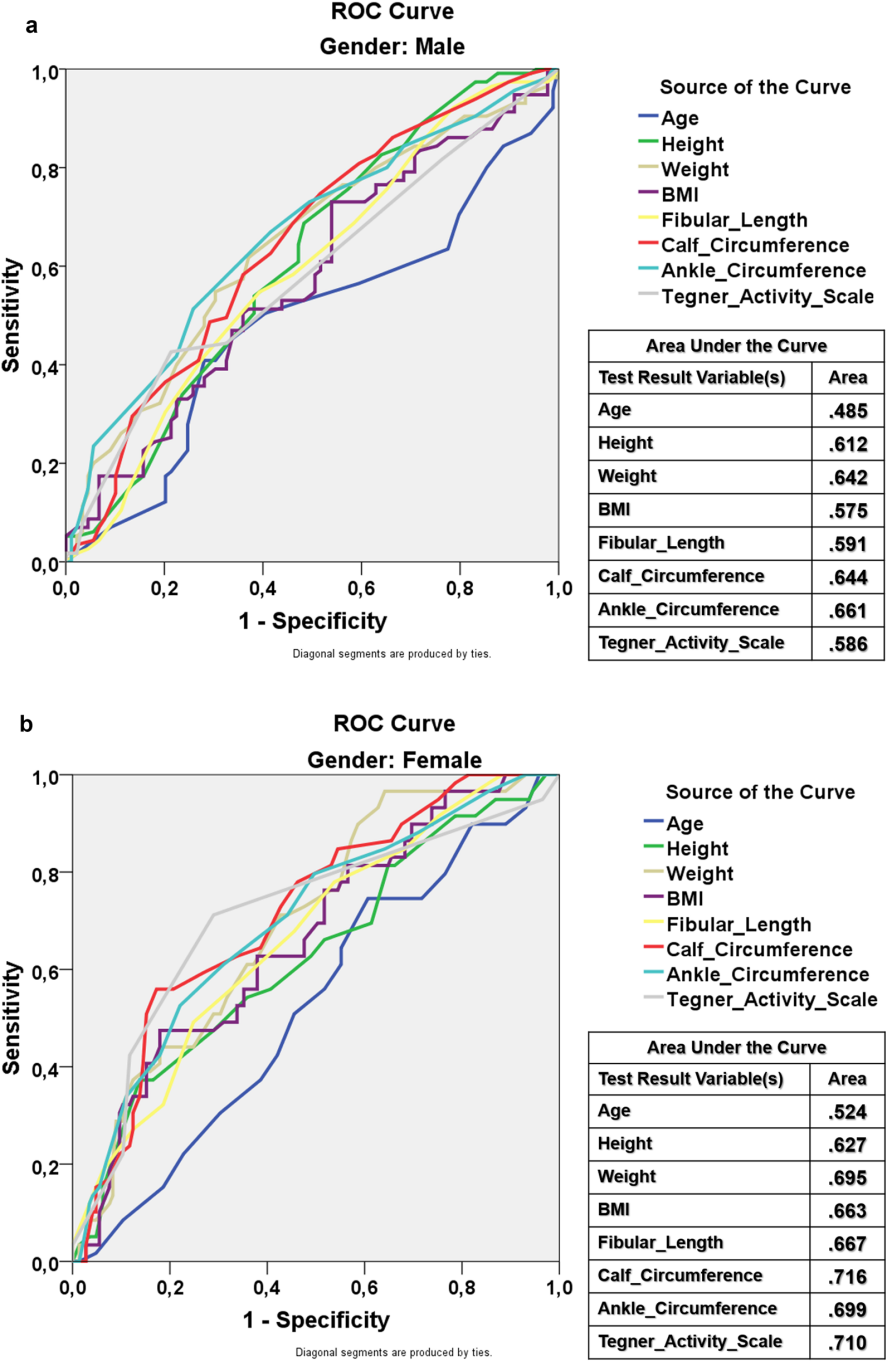
ROC curves demonstrating the discriminatory abilities of anthropometric variables in predicting sufficient PLT graft thickness (≥ 8 mm) across genders. Each curve is color-coded for clarity, with enhanced contrast and simplified legends to facilitate easier interpretation. The area under the curve (AUC) values for each variable are explicitly noted to highlight their respective predictive capabilities

ROC analysis showed that, in males, none of the demographic or anthropometric variables had an AUC above the acceptable limit (AUC > 0.700). In females, calf circumference and the Tegner Activity Scale reached acceptable discriminatory abilities (Fig. 4). The optimal cutoff points were 36.25 cm for calf circumference (sensitivity of 78% and a specificity of 53.8%) and ≥ 4 for the Tegner Activity Scale (71.2% and a specificity of 71.0%).

## Discussion

The most significant finding of this study is that calf circumference and the Tegner Activity Scale are strong predictors of adequate two-stranded PLT graft thickness in females, potentially improving preoperative planning for ACLR. Males had significantly thicker grafts than females, consistent with previous studies on gender-specific tendon dimensions. Notably, 43.6% of males and 70% of females had graft thicknesses below the 8 mm threshold, traditionally considered insufficient for ACLR, highlighting the need for preoperative assessments, especially in females. Augmentation techniques may be necessary to achieve sufficient graft thickness during surgery. Binary logistic regression identified height and calf circumference as predictors of graft thickness in males, while calf circumference and the Tegner Activity Scale were key predictors in females. However, ROC analysis showed poor predictive power for males but moderate discriminatory ability for females, with cutoff points of 36.25 cm for calf circumference and ≥ 4 for the Tegner Activity Scale, offering practical guidelines to identify female patients at risk of insufficient graft thickness.

This study showed significant differences in PLT graft thickness between males and females, with males having thicker grafts. Although partly explained by greater height and weight in males, the difference persisted even after adjustment for these factors, suggesting that physiological characteristics, such as greater muscle mass, contribute to larger tendon size. In females, higher Tegner activity levels significantly predicted tendon thickness, supporting the idea that physical activity promotes muscle and tendon hypertrophy. These findings align with previous studies on gender-specific variations in PLT graft thickness [3–5, 8, 9, 13, 14]. Identifying female patients with insufficient tendon thickness is critical since grafts under 8 mm are linked to higher ACLR failure risk. Song et al. reported that 36% of female patients had grafts below 8 mm using a four-stranded PLT [14], while Sakti et al. [8] and Ertilav [9] found all female patients in their studies had grafts under 8 mm. In contrast, Khan et al. observed no grafts below 8.5 mm using a distal harvest site for a four-stranded graft, suggesting that distal harvesting may enhance PLT graft thickness and strength [13].

Another key finding is that calf circumference significantly predicted PLT graft thickness in both genders, with stronger predictive power in females. This aligns with Ertilav [9] and Wierer et al. [12], who highlighted the importance of localized anthropometric measurements for predicting graft size. Ertilav [9] found that distal leg diameter had the highest correlation with PLT graft diameter (*r* = 0.956), emphasizing the role of calf circumference in predicting graft thickness. Wierer et al. [12] showed that graft length was most strongly predicted by height, while graft diameter moderately correlated with weight. These findings further support the use of calf circumference in preoperative assessments, particularly for females, to improve graft size predictions.

Additionally, activity levels in females were found to predict PLT graft thickness. Song et al. [14] explored this relationship in a retrospective study of 156 ACLR patients and found correlations between graft diameter and factors such as height, weight, and injury duration, but not preinjury activity levels. The lack of correlation was attributed to the self-reported nature of activity levels, which can introduce bias. In our study, activity levels were also self-reported, but our participants were healthy, whereas Song et al.’s patients likely experienced muscle atrophy due to prolonged immobilization after ACL injury. Song et al. [14] also noted thinner PLT grafts in patients with ACL ruptures longer than 3 months, likely due to disuse atrophy.

This study has several strengths and limitations. One limitation is the use of ultrasonographic measurements to estimate PLT graft thickness, which may introduce inaccuracies compared with direct intraoperative measurements. However, Leu et al. reported that the margin of error for this method was less than 0.5 mm in 96.5% of cases [11]. Another limitation is that the study was conducted at a single institution with a Caucasian sample, which may limit generalizability to other populations. Despite these limitations, the large, gender-balanced sample size improves the reliability of the findings. Additionally, the study did not include graft length data, an important parameter in ACLR.

## Conclusions

Calf circumference and the Tegner Activity Scale are significant predictors of two-stranded PLT graft thickness in females. These thresholds offer practical value in ACLR preoperative planning. However, no anthropometric variables were identified as strong predictors of graft thickness in males.

## Data Availability

The datasets generated during and/or analyzed during the current study are available from the corresponding author upon reasonable request.

## Abbreviations

PLT: Peroneus longus tendon
PBT: Peroneus brevis tendon
ACL: Anterior cruciate ligament
ACLR: Anterior cruciate ligament reconstruction
BMI: Body mass index
CSA: Cross-sectional area
USG: Ultrasonography
ROC: Receiver operating characteristic
TAS: Tegner Activity Scale
